# Trait‐based evidence of salinity‐induced functional diversity loss in mangroves: Implications for ecosystem resilience

**DOI:** 10.1002/eap.70191

**Published:** 2026-02-24

**Authors:** Md Rezaul Karim, Nabanita Karmaker, Shekhar R. Biswas, Md. Shamim Reza Saimun, Sharif A. Mukul, Tanjena Khatun, Fahmida Sultana, Sanjeev K. Srivastava, Mohammed A. S. Arfin‐Khan

**Affiliations:** ^1^ Institute of Forestry and Conservation, John H. Daniels Faculty of Architecture, Landscape, and Design University of Toronto Toronto Ontario Canada; ^2^ Department of Forestry and Environmental Science School of Agriculture and Mineral Sciences, Shahjalal University of Science and Technology Sylhet Bangladesh; ^3^ Zhejiang Zhoushan Archipelago Observation and Research Station, National Observation and Research Station of the Tiantong Forest Ecosystem, and Shanghai Key Laboratory for Urban Ecological Processes and Eco‐Restoration School of Ecological and Environmental Sciences, East China Normal University Shanghai China; ^4^ Department of Environment and Development Studies United International University Dhaka Bangladesh; ^5^ Department of Earth and Environment Florida International University Miami Florida USA; ^6^ Department of Biological Science University of Quebec in Montreal (UQAM) Montreal Quebec Canada; ^7^ School of Biosciences, Geography and Physics, Faculty of Science and Engineering Swansea University Swansea Wales; ^8^ School of Science Technology and Engineering (SSTE), University of the Sunshine Coast Maroochydore DC Queensland Australia

**Keywords:** functional diversity, functional traits, mangrove ecology, salinity gradient, Sundarbans, trait convergence

## Abstract

Mangrove forests—vital for global carbon storage and coastal protection—are increasingly threatened by salinity intrusion resulting from sea‐level rise and alterations in the hydrological regimes. While the functional importance of mangroves is well recognized, the mechanistic pathways through which salinity reorganizes community‐level trait composition and compresses functional diversity remain unresolved. This gap is particularly acute in megadeltaic systems like the Sundarbans, where biodiversity and ecosystem service provisioning co‐occur with steep salinity gradients. Elucidating how trait syndromes shift and diversity contracts across these gradients is critical to forecasting mangrove ecosystem responses and informing adaptive conservation strategies. This study quantified eight foliar traits (leaf area, specific leaf area, leaf dry matter content, total chlorophyll, stomatal density, leaf shape index, leaf succulence, and leaf carbon content) and four functional diversity indices (Rao's quadratic entropy, functional richness, evenness, and divergence) across a continuous soil salinity gradient using plot‐level data from 59 sites in the Sundarbans. Trait–environment relationships were analyzed using linear regressions, spatial mapping, and multivariate ordination (principal components analysis [PCA], non‐metric multidimensional scaling [NMDS]), while controlling for biotic factors such as species richness and abundance. Salinity significantly reduced functional diversity, particularly trait dissimilarity (RaoQ), supporting the hypothesis of abiotic filtering that favors functionally similar, salt‐tolerant species. These reductions were most pronounced in high‐salinity western zones dominated by generalist stress‐tolerant species. Foliar traits shifted predictably with salinity, with reductions in leaf area, dry matter content, stomatal density, chlorophyll, and carbon content, and increases in leaf succulence and specific leaf area—indicating trade‐offs toward conservative resource‐use strategies under osmotic stress. Species abundance strongly influenced functional diversity independent of salinity. High abundance reduced trait dissimilarity and evenness, reinforcing the dominance of a few trait syndromes under stress. By integrating spatially explicit trait, salinity, and abundance data, this study provides novel evidence that abiotic filtering and biotic dominance jointly constrain community‐level functional diversity in mangroves. Trait convergence and dissimilarity collapse under salinity stress indicate narrowing ecological strategies with reduced resilience. Conservation strategies should prioritize freshwater inflow and low‐salinity habitat restoration. Trait‐based indicators offer a predictive framework to sustain mangrove function under accelerating climate stress.

## INTRODUCTION

Mangrove forests are globally significant coastal ecosystems that provide high productivity, carbon sequestration, and coastal protection (Arceo‐Carranza et al., 2021; Karim et al., 2025; Sunkur et al., 2023). Despite their importance, mangroves are threatened by anthropogenic and climatic pressures, including altered freshwater inflows and rising sea levels, which intensify soil salinity (Bhowmik et al., 2022; Padhy et al., 2022). Salinity acts as a primary ecological filter, shaping plant physiology, growth, and species distribution by affecting osmotic potential, nutrient uptake, and ion toxicity (Ahmed et al., 2022; Meera et al., 2023).

These effects are especially pronounced in estuarine systems, where tidal influx, freshwater discharge, and evapotranspiration generate complex spatiotemporal salinity gradients (Atekwana et al., 2022; Wang, Xin, et al., 2024). Such gradients impose selective pressures that favor stress‐tolerant species with traits suited to prevailing conditions, making mangroves ideal for examining how environmental stress governs community assembly and ecosystem functioning (Busoms et al., 2023). While species zonation along salinity gradients is well documented (Bathmann et al., 2021; Chen & Twilley, 1998; Liu et al., 2024), the mechanisms linking continuous salinity variation to shifts in community‐level foliar traits and functional diversity (FD) remain empirically underexplored, limiting our ability to predict the resilience of a vital ecosystem.

In recent decades, trait‐based ecology has emerged as a powerful framework to investigate how environmental filters modulate community structure and functioning. Foliar traits such as specific leaf area (SLA), leaf dry matter content (LDMC), succulence, and stomatal density (SD) capture plant resource‐use strategies and physiological trade‐offs under stress, including drought and salinity (Hočevar et al., 2025; Wang et al., 2011). FD metrics quantify the range, distribution, and dissimilarity of these traits in a community. Key FD metrics such as functional richness (FRic), evenness (FEve), divergence (FDiv), and Rao's quadratic entropy (Rao's *Q*) provide mechanistic insights to evaluate how abiotic filters and competitive interactions shape trait dispersion and community resilience (Li et al., 2024; Rocchini et al., 2021; Zhang et al., 2021).

Empirical evidence from stress‐prone ecosystems suggests that increasing abiotic stress, such as salinity or aridity, can lead to reduced FD through trait convergence (Gong et al., 2019). In such environments, selection tends to favor stress‐tolerant species that exhibit similar trait syndromes, defined as recurring sets of functional traits that jointly reflect adaptive responses to ecological pressures. These syndromes are typically characterized by conservative resource‐use strategies, robust tissue construction, and lower metabolic rates (Perri et al., 2023). For instance, mangrove species in hypersaline zones often exhibit high LDMC, thickened leaves, and reduced SLA, traits that minimize transpirational water loss and confer mechanical and osmotic resilience (Meera et al., 2023). These trait filters are expected to reduce the volume and complexity of occupied functional space, thereby lowering FD and potentially limiting ecosystem adaptability. Yet, the extent to which such functional filtering occurs along continuous salinity gradients—especially in real‐world mangrove ecosystems with spatial heterogeneity and demographic stochasticity—remains inadequately explored.

Adding to this uncertainty is the confounding role of biotic structure—particularly species richness and relative abundance—in shaping FD metrics. Richness can inflate FRic simply by increasing the number of trait combinations represented in a community, while species dominance can skew community‐weighted trait means (Muscarella & Uriarte, 2016). Thus, without proper statistical controls, trait–environment relationships may be misattributed to abiotic filtering. This challenge has been recognized in trait‐based studies across multiple biomes, including temperate grasslands (Roscher et al., 2013) and tropical forests (Lebrija‐Trejos et al., 2010), yet remains largely unexplored in mangrove ecosystems, where functional studies are sparse and often lack the methodological rigor to disentangle abiotic and biotic influences.

The Sundarbans, the world's largest continuous mangrove forest spanning Bangladesh and India, provides a compelling natural setting to investigate these dynamics. Its well‐characterized salinity gradient arises from complex interactions among riverine freshwater inflow, estuarine mixing, and tidal intrusion (Akbar Hossain et al., 2021), which establishes three distinct salinity zones (Sarker et al., 2019). These environmental gradients influence species zonation, biomass distribution, and habitat suitability across the landscape (Sarker et al., 2024); yet, the functional mechanisms underlying these patterns remain unclear. Previous studies have examined species‐level functional trait and plant type variation across salinity zones (e.g., Ahmed et al., 2022; Khan et al., 2020; Rahman, 2020; Sarker et al., 2024) and documented community‐level shifts (Dasgupta et al., 2017), but integrated community‐level analyses combining foliar trait composition, community structure, and fine‐scale salinity are rare. Such integration is critical because trait–environment relationships are strongly scale‐dependent and can be obscured when investigated at coarse resolutions. Furthermore, studies that neglect species richness and abundance risk conflating abiotic filtering with biotic assembly, thereby limiting inference about ecological processes.

Trait‐based approaches are increasingly being used for restoration, conservation, and carbon sequestration initiatives in mangroves (Loureiro et al., 2023); yet, current applications are limited by the lack of mechanistically grounded, spatially explicit, and statistically robust empirical evidence. Addressing this gap is essential not only for advancing theoretical ecology but also for developing evidence‐based management strategies in the face of escalating climate stress.

In the present study, we investigate how FD and foliar trait composition vary along a continuous soil salinity gradient in the Sundarbans mangrove forest. Our goal is to disentangle the effects of abiotic filtering, that is, the process by which environmental conditions select for species with traits suited to prevailing stresses, from those arising due to biotic structure—particularly species richness and abundance—which may independently influence trait dispersion. This dual focus is motivated by the recognition that salinity not only imposes direct physiological stress but also drives species turnover, leading to shifts in community composition and ecological strategy (Röthig et al., 2023). As a result, we expect salinity‐driven abiotic filtering to reduce trait dispersion and thereby FD. At the same time, biotic factors such as high species richness or dominance can increase trait variability through competitive niche differentiation or simply expand the occupied trait space. Ignoring such biotic influences may obscure or exaggerate the effects of salinity on trait‐based community assembly.

Based on this reasoning, we propose three interrelated hypotheses: (i) FD (FRic, FEve, FDiv, and Rao's *Q*) decreases with increasing soil salinity due to trait convergence under stress, as salinity filters out functionally dissimilar species; (ii) community‐weighted means (CWMs) of key foliar traits—specifically, increased SLA, reduced LDMC, and increased succulence—are expected in more saline plots, reflecting physiological adaptations to osmotic stress; and (iii) species richness and abundance modulate observed FD metrics by altering trait space independently of salinity, and thus must be explicitly controlled to avoid confounded inferences. Collectively, these hypotheses are designed to examine empirical patterns of trait variation and community assembly processes in mangrove ecosystems, without implying the use of mechanistic models or fully integrated trait–environment frameworks. The study thus seeks to provide context‐specific evidence on salinity–trait relationships that may inform restoration planning and conservation strategies in the Sundarbans, while contributing to ongoing discussions in trait‐based ecology.

## METHODS

### Study site

The Sundarbans, the largest mangrove forest globally, spans the northern Bay of Bengal across the India–Bangladesh border. The Bangladeshi section, between 89°00′ 9°55′ E, 21°30′–22°30′ N, covers approximately 599,330 ha (62% of the forest), while the remaining 426,300 ha are in West Bengal, India (Borrell et al., 2016). Renowned for its biodiversity and productivity, the Sundarbans buffer against cyclones, tidal flooding, and coastal erosion (Borrell et al., 2016; Payo et al., 2016). It acted as a protective barrier during cyclones Sidr (2007), Aila (2009), and Amphan (2020) (Mishra et al., 2021).

The Sundarbans consist of around 200 islands intersected by over 400 tidal rivers and canals (Sen & Ghorai, 2019). The forest hosts 334 plant species, including 50 mangrove species alongside legumes, grasses, sedges, and euphorbias (Chaffey et al., 1985). Key species include *Heritiera fomes* (Sundari), *Ceriops decandra* (Goran), *Excoecaria agallocha* (Gewa), *Nypa fruticans* (Golpata), *Sonneratia apetala* (Keora), *Xylocarpus mekongensis* (Passur), *Avicennia officinalis* (Baen), and *Rhizophora mucronata* (Garjan). *H. fomes* is particularly threatened by top‐dying disease (Islam et al., 2014), while rare species like *Bruguiera parviflora* (Bhat Kati) and *Avicennia marina* (Kala Baen) are also present in this ecosystem (Islam et al., 2014). The Sundarbans provide critical habitat for a wide array of fauna, including 50 mammal species, 320 bird species, 53 reptile species, 11 amphibian species, 177 fish species, and 873 invertebrate species (Mukul et al., 2019). The Bangladeshi Sundarbans alone support 45% of the nation's mammal species, 42% of bird species, 46% of reptiles, and 36% of amphibians (Ahmed & Rahman, 2023).

### Sampling design and data collection

**FIGURE 1:**
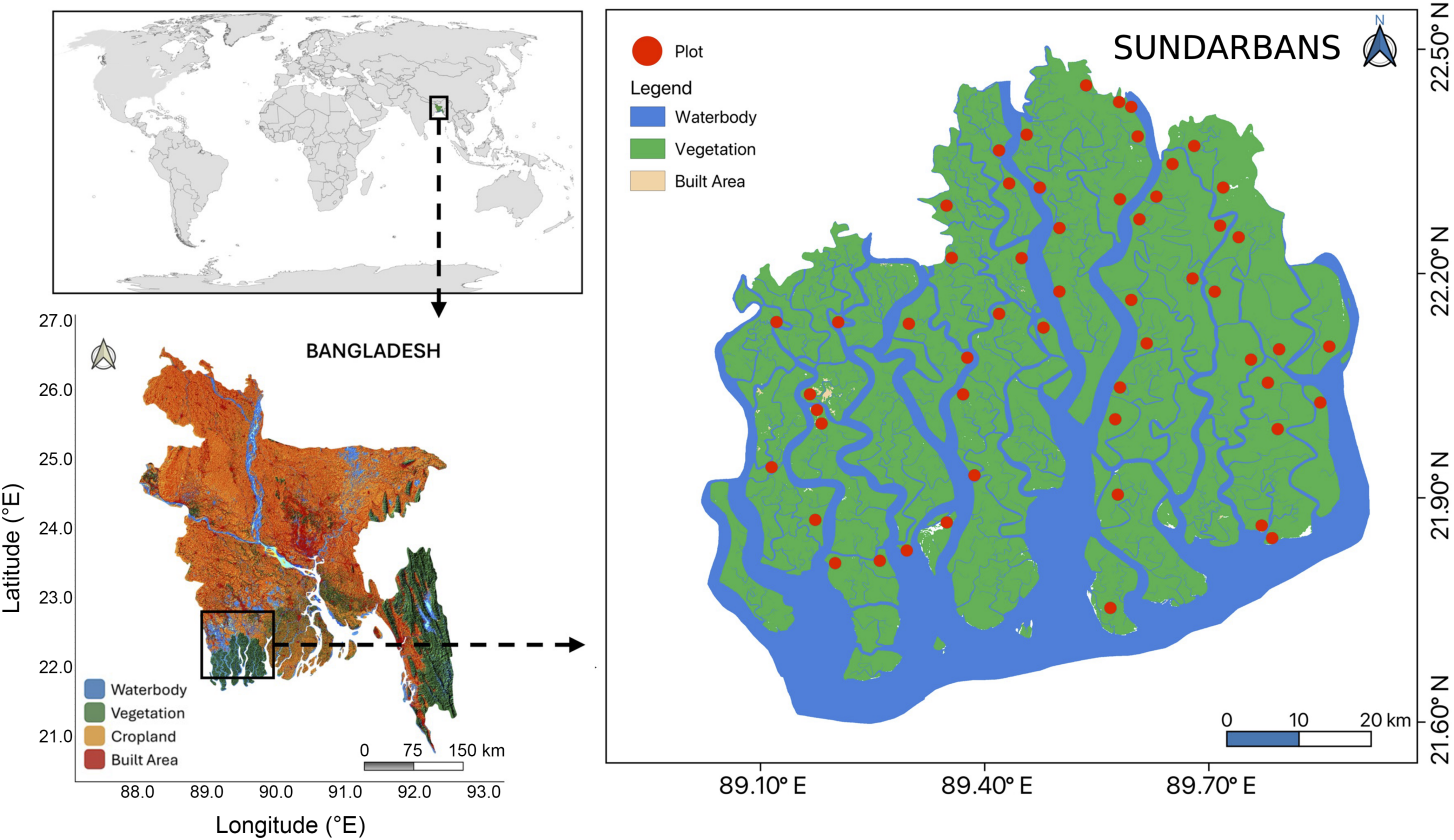
Map of the study area in the Sundarbans, Bangladesh. The red circle indicates the location of the study plot.

Vegetation, foliar trait, and soil data were collected from 62 georeferenced sampling plots, each measuring 20 m × 20 m, distributed across all 54 management compartments of the Sundarbans mangrove forest (Figure 1). Plots were selected to capture spatial heterogeneity in salinity and elevation while minimizing edge effects by maintaining a minimum distance of 120 m from any water channel or river. Data collection was conducted between October and March of the 2021–2022 dry season, a period chosen to minimize confounding seasonal variability in foliar and soil traits while ensuring safe and consistent field access. This window offered stable hydrological conditions, reduced cyclone and insect disturbance, and foliage in full mature growth status, thereby providing the most reliable representation of community‐level trait variation across the landscape. Plot locations and elevations were recorded using a handheld GPS unit (GPSMAP‐65, Garmin Ltd., Olathe, KS, USA).

### Vegetation data collection

Within each sampling plot, all trees were identified to their appropriate taxonomic identity using local expert knowledge, as well as references such as the Encyclopedia of Flora and Fauna of Bangladesh (Ahmed, 2008a, 2008b, 2009a, 2009b). A total of 17 distinct tree species were identified across the study plots (Table 1). Trees with a dbh of ≥5 cm (measured at 1.3 m above ground) were included in the analysis. Tree height was determined using a Haga altimeter (Haga GMBH & Co., Nuremberg, Germany) by using the following formula:

$$\begin{array}{l} \text{Tree height}\\ \;=\frac{(\text{top crown reading}-\text{bottom reading})\times \text{ground distance}}{100}\\ +1.6\;\text{m} \end{array}$$

**TABLE 1:** List of species that have been assessed in this study.

Local name	Scientific name	Habitat type
Amoor	*Aglaia cucullata*	Low‐saline zone
Batul	*Excoecaria indica*	Low‐saline zone
Dhakur	*Cerbera odollum*	Low‐saline zone
Dhundul	*Xylocarpus granatum*	Mid‐saline zone
Garjan	*Rhizophora apiculata*	High‐saline zone
Gewa	*Excoecaria agallocha*	Low‐saline zone, mid‐saline zone
Goran	*Ceriops decandra*	Mid‐saline zone, high‐saline zone
Kakra	*Bruguiera sexangula*	Mid‐saline zone
Kala Bean	*Avicennia officinalis*	High‐saline zone
Keora	*Sonneratia apetala*	Mid‐saline zone
Khulshi	*Aegiceras corniculatum*	Mid‐saline zone
Lal Kakra	*Bruguiera gymnorrhiza*	High‐saline zone
Passur	*Xylocarpus moluccensis*	Mid‐saline zone
Rohini	*Kandelia candel*	Mid‐saline zone
Sada Bean	*Avicennia alba*	High‐saline zone
Singra	*Cynometra ramiflora*	Low‐saline zone
Sundari	*Heritiera fomes*	Low‐saline zone

We used meter tape to measure the ground distance.

Tree abundance was estimated by conducting a direct count of individuals within each plot. The collected species list has been provided in Table 1.

### Functional trait data collection

We selected eight key foliar traits indicative of salinity tolerance, water‐use efficiency, and stress response: leaf area (LA, in square centimeters), SLA (in square centimeters per gram), LDMC (in milligrams per milligram), total chlorophyll content (TC, in milligrams per gram), SD (in number of stomata per square millimeter), leaf shape index (LSI, dimensionless), leaf succulence (LS, in grams per square centimeter), and leaf carbon content (LC, in percentage of dry mass) (Table 2). From each species, 3–10 mature leaves were collected, with sampling randomized across accessible individuals. Because large areas of the plots were difficult to access, it was not always possible to obtain samples from all three salinity zones for the same species; nevertheless, we sought to balance representation across zones whenever feasible. Leaves were collected from sun‐exposed positions in the mid‐ to lower canopy. Immediately after collection, samples were sealed in sterile zip‐lock bags, stored in a refrigerator on the launch‐boat (~−7°C), and transported to the laboratory on ice packs. Leaves were scanned or measured with the Petiole mobile app to determine fresh area (Singh et al., 2021). Green mass (LGM) was weighed with a digital balance, followed by oven‐drying at 80°C for 48 h to obtain dry mass (LDM). LDMC was calculated from fresh and dry mass (Garnier et al., 2001). SD was determined using the nail polish imprint technique on the abaxial surface. Total chlorophyll content was recorded using the standard method in Ritchie (2008). LC was measured via dry combustion in a muffle furnace and calculated as Equation (1) and then Equation (2) (Shaw, 1959):

(1) $$\mathrm{Ash}\;\left(\%\right)=\frac{W_{3}-W_{1}}{W_{2}-W_{1}}\times 100$$

then,

(2) $$C\left(\%\right)=\left(100-\mathrm{Ash}\%\right)\times 0.58$$

where *W*_1_ = crucible weight, *W*_2_ = leaf + crucible, *W*_3_ = ash + crucible. Species‐level means were computed from all leaf‐level observations. A detailed description of the eight measured foliar traits, including formulas and references, is provided in Appendix S1: Table S1.

**TABLE 2:** Replication statement.

Scale of inference	Scale at which the factor of interest is applied	Factor	Level	No. replicates
Community‐level functional diversity	Plot	Soil salinity (EC)	Continuous (in situ EC measurements averaged across 5 depth layers per plot)	59 plots (≥3 functionally distinct species)
Community‐level trait composition (CWMs)	Species within plots	Species abundance and identity	Relative abundance (plot‐level)	62 plots (total), 59 used in FD analysis
Trait–environment relationships (CWMs)	Species (within plots)	Foliar traits	Leaf area, SLA, LDMC, LS, LSI, SD, TC, LC	8 traits × 17 species × 3–10 leaves per species per plot
Functional diversity indices (RaoQ, FRic, FEve, FDiv)	Plot (trait matrix weighted by abundance)	Multivariate trait matrix	8‐trait Gower distance matrix	59 plots (plots with <3 species excluded for FRic/FEve/FDiv)
Soil covariates	Plot	Elevation, pH, bulk density, total N	Measured in each plot	59 plots

Abbreviations: CWMs, community‐weighted means; EC, electrical conductivity; FD, functional diversity; FDiv, functional divergence; FEve, functional evenness; FRic, functional richness; LC, leaf carbon content; LDMC, leaf dry matter content; LS, leaf succulence; LSI, leaf shape index; RaoQ, Rao's quadratic entropy; SD, stomatal density; SLA, specific leaf area; TC, total chlorophyll content.

### Trait scaling and CWM calculation

CWMs for each trait were calculated at the plot level using species‐level average trait values and relative abundances. For instance, if Plot 2 contained 4 individuals of *H. fomes* and 2 of *E. agallocha*, the CWM for a given trait was computed as Equation (3):

(3) $${\mathrm{CWM}}_{\text{trait}}=\frac{\left(4\times {\text{Trait}}_{\mathrm{Hf}}+2\times {\text{Trait}}_{\mathrm{Ea}}\right)}{6}$$

Species presence and abundance data from each plot were used to compute relative weights. This approach ensured that trait distributions reflected both species composition and local dominance patterns.

### Soil data collection and analysis

Soil samples were collected from two subplots within each main plot to a depth of 100 cm, subdivided into five layers: 0–10 cm, 11–20 cm, 21–30 cm, 31–50 cm, and 51–100 cm. For each depth layer, soil from the two subplots of a given plot was combined to create a composite sample for that specific layer, ensuring that within‐plot spatial variability was minimized while preserving vertical stratification. Soil salinity was assessed in the Soil Science Laboratory at the Department of Forestry and Environmental Science, Shahjalal University of Science and Technology, Bangladesh. Electrical conductivity (EC) was measured using a portable pH/EC/TDS meter (Model HI9810‐61, Hanna Instruments, USA). A soil‐to‐distilled water ratio of 1:2 was employed, and soil dilution was used to ensure accurate measurements, following the protocol outlined by Monteleone et al. (2016). The EC values for each of the five depth layers were averaged to obtain a representative measure of soil salinity (in millisiemens per centimeter) for further analysis. Additional soil parameters were measured using standard protocols: elevation (in meters) was recorded using a Hanna handheld GPS meter, soil pH was determined using a calibrated portable pH meter (Model HI9810‐61, Hanna Instruments), bulk density (in grams per cubic centimeter) was measured from oven‐dried core samples following gravimetric methods, and total nitrogen (in percentage) was assessed via the Kjeldahl digestion method (Kirk, 1950).

### Functional diversity measurement

#### Community weighted mean

CWM quantifies the functional composition of a community by averaging trait values weighted by species abundance. The CWM for a given trait *t* is calculated using the formula (Equation 4):

(4) $${\mathrm{CWM}}_{t}=\sum_{i=1}^{S}p_{i}\times t_{i}$$

where $t_{I}$ represents the trait value for species *I*, $p_{I}$ denotes the relative abundance of species *I*, and *S* is the total number of species in the community. This index reflects the average trait value across the community, weighted by species presence (Violle et al., 2007).

#### Quadratic entropy of Rao (RaoQ)

RaoQ is a measure of FD that incorporates both species' relative abundances and the pairwise functional differences among species (Botta‐Dukát, 2005). It was calculated using Equation (5):

(5) $${\mathrm{FD}}_{Q}=\sum_{i=1}^{S-1}\sum_{j=i+1}^{S}d_{\mathrm{ij}}\times p_{i}\times p_{j}$$

where $d_{\mathrm{ij}}$ denotes the functional difference between species *I* and *j*, and $p_{I}$ and $p_{j}$ represent the relative abundances of species *I* and *j*, respectively. RaoQ quantifies the average functional dissimilarity between pairs of species within the community.

#### Functional richness (FRic)

FRic quantifies the volume of functional trait space occupied by the species within a community, based on the convex hull that encompasses all trait combinations (Legras et al., 2018). It reflects the range of trait values but does not account for species abundance. FRic is sensitive to outlier trait values and requires at least three functionally distinct species per plot.

#### Functional evenness (FEve)

FEve measures the regularity with which species abundances are distributed across the functional trait space (Biswas et al., 2016). High FEve indicates an even spacing and abundance of species, while low FEve suggests trait clustering or dominance by a few functional types. This metric uses minimum spanning trees to assess how well trait space is filled.

#### Functional divergence (FDiv)

FDiv assesses how species abundances diverge from the centroid of the functional space, highlighting the degree to which abundant species possess extreme or peripheral trait values (Mouillot et al., 2005). High FDiv suggests niche differentiation and resource partitioning, whereas low values imply redundancy or functional centralization.

#### FD estimation

FD was quantified using five indices: FRic, FEve, FDiv, Rao's quadratic entropy (RaoQ), and functional dispersion (FDis). All indices were calculated using the dbFD() function from the FD R package (Magneville et al., 2022). Trait dissimilarities were computed using Gower distance based on the eight standardized foliar traits. Trait values were scaled to zero mean and unit variance prior to distance calculation. A Cailliez correction was applied to correct for negative eigenvalues in the dissimilarity matrix (Caillez & Kuntz, 1996). To meet the *s* > *t* condition for FRic, two principal component analysis axes were retained (out of an initial eight), and the resulting functional space had a quality of 0.625. Plots with fewer than three functionally distinct species were excluded from FRic, FEve, and FDiv calculations, resulting in a final dataset of 59 plots for those indices.

### Statistical analysis

The scale of inference, number of replicates, and levels for each factor are presented in the Replication statement (Table 2). Analyses were conducted in R 4.4.1 (R Core Team, 2024) and Python 3.11.0. A combination of exploratory, multivariate, spatial, and regression‐based approaches was used to test three hypotheses: (a) FD declines with salinity (trait convergence), (b) foliar trait composition shifts along salinity gradients, and (c) species richness and abundance independently influence FD metrics.

Trait interrelationships were examined using Pearson correlations and density plots (*psych*, *GGally*), providing preliminary support for (b) by identifying associations among SLA, LDMC, and succulence. PCA on standardized CWM traits further addressed (b) by visualizing axes of trait variation linked to salinity. Community composition was explored with non‐metric multidimensional scaling (NMDS) based on Bray–Curtis dissimilarities, addressing (c) by evaluating how species richness and abundance structured trait dispersion. FD indices (FRic, FEve, FDiv, RaoQ, and FDis) and CWMs were modeled using linear regressions against environmental (EC, elevation, pH, bulk density, and total N) and biotic predictors (species richness and abundance). These regressions tested (a) salinity‐driven declines in FD and (c) independent biotic effects.

To integrate spatial patterns, bivariate choropleth maps (*biscale*, *sf*, and *cowplot*) of EC and FD metrics were generated, contextualizing (a) and (b) by visualizing salinity–trait co‐variation across the Sundarbans. Figures were prepared with *ggplot2*, *patchwork*, *matplotlib*, and *seaborn*.

## RESULTS

### FD responses to soil salinity

**FIGURE 2:**
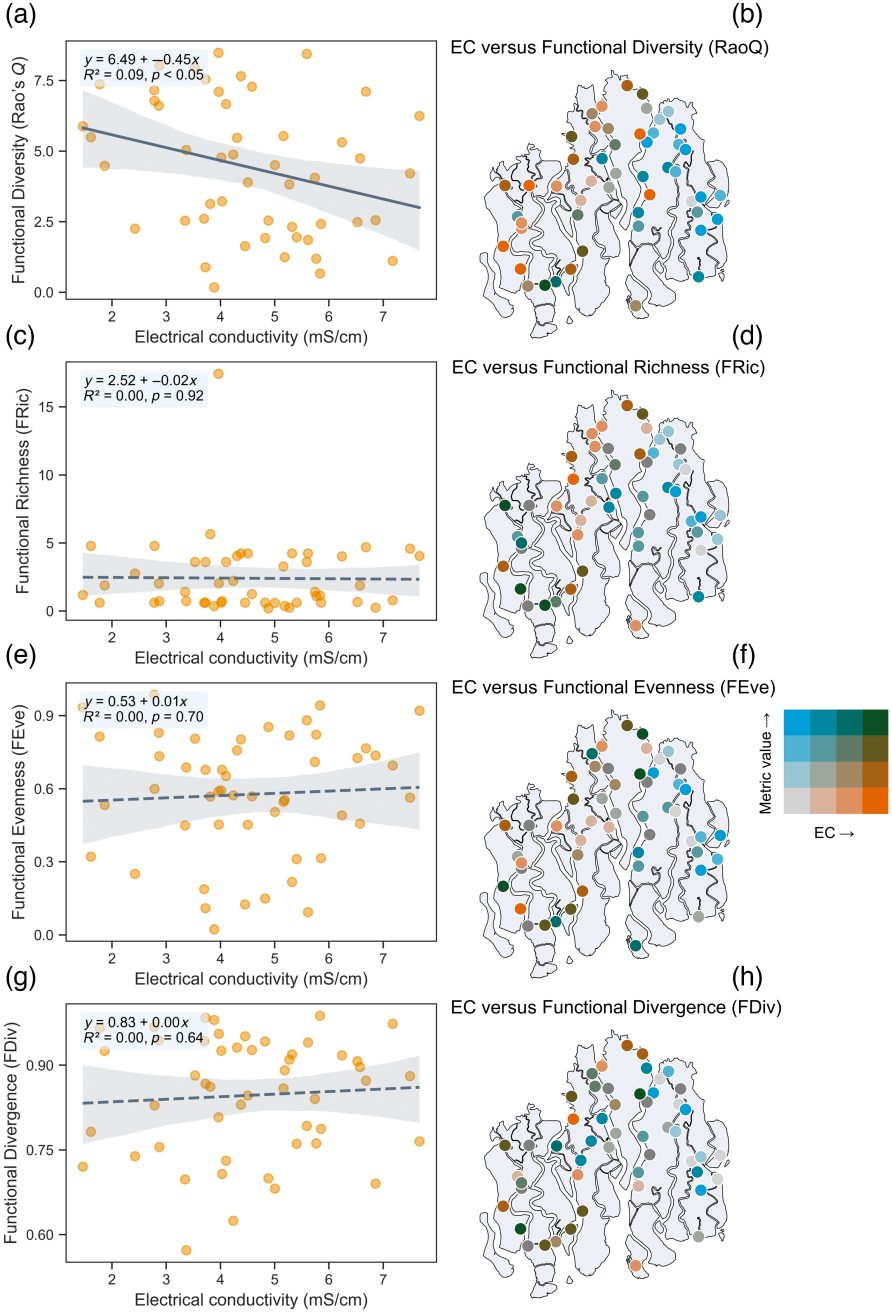
Relationships between electrical conductivity (EC) and four functional diversity metrics across 59 mangrove forest plots in the Sundarbans. Panels (a), (c), (e), and g show linear regressions of EC against functional diversity (Rao's quadratic entropy), functional richness, functional evenness, and functional divergence, respectively. Panels (b), (d), (f), and (h) depict the spatial distribution of each bivariate relationship based on quantile classification of EC and the corresponding functional metric.

Among the four measured FD indices, only Rao's quadratic entropy (RaoQ) exhibited a significant relationship with soil salinity (Figure 2a–h). Specifically, RaoQ declined modestly but significantly with increasing EC, indicating a reduction in trait dissimilarity under high‐salinity conditions (slope = −0.45, *R* ^2^ = 0.09, *p* < 0.05; Figure 2a). In contrast, FRic, FEve, and FDiv showed no significant associations with EC (FRic: slope = −0.02, *R* ^2^ < 0.01, *p* = 0.92; FEve: slope = 0.01, *R* ^2^ < 0.01, *p* = 0.70; FDiv: slope = 0.00, *R* ^2^ < 0.01, *p* = 0.64; Figure 2c,e,g). Spatial bivariate maps revealed clear spatial clustering of FD metrics across the salinity gradient, with lower RaoQ values concentrated in the western, more saline zones of the Sundarbans (Figure 2b,d,f,h).

### Influence of biotic and edaphic variables on FD

**FIGURE 3:**
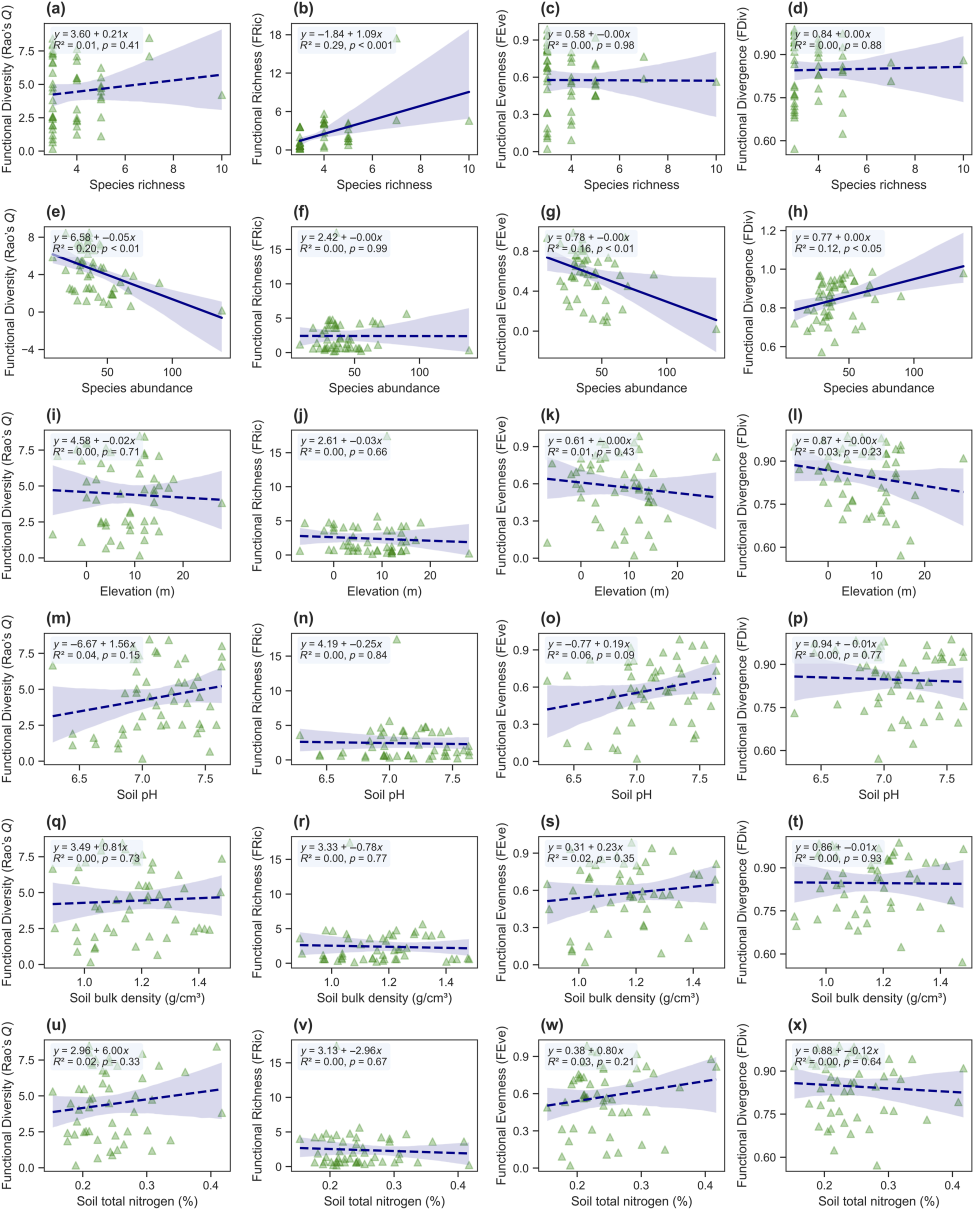
Bivariate relationships between functional diversity metrics and environmental predictors. Each column represents a functional diversity metric: Functional diversity (Rao's *Q*), functional richness (FRic), functional evenness (FEve), and functional divergence (FDiv). Each row corresponds to a predictor: Species richness, species abundance, elevation, soil pH, soil bulk density, and soil total nitrogen. Regression equations, coefficients of determination (*R* ^2^), and *p* values are annotated in each panel. Line styles denote significance: Solid for *p* ≤ 0.05 and dashed for *p* > 0.05.

FD metrics showed varying degrees of sensitivity to biotic and edaphic predictors beyond salinity (Figure 3a–x). Among biotic drivers, species abundance exerted the strongest and most consistent effects. RaoQ decreased significantly with increasing abundance (slope = −0.05, *R* ^2^ = 0.20, *p* = 0.001), suggesting that highly abundant communities exhibit lower functional dissimilarity, potentially due to trait dominance by a few stress‐tolerant species (Figure 3e). Abundance also negatively predicted FEve (slope = −0.005, *R* ^2^ = 0.16, *p* = 0.004) and positively predicted FDiv (slope = 0.002, *R* ^2^ = 0.12, *p* = 0.014), indicating potential shifts in the distribution and extremity of traits under dense conditions (Figure 3g,h). Species richness, by contrast, was a strong predictor of FRic (slope = 1.09, *R* ^2^ = 0.29, *p* < 0.001), supporting the expectation that trait space expands with higher taxonomic diversity (Figure 3b). Other FD metrics showed no significant associations with richness.

Environmental variables showed limited explanatory power. Among edaphic predictors, soil pH exhibited a marginally significant positive effect on FEve (slope = 0.19, *R* ^2^ = 0.06, *p* = 0.09), while other FD indices remained statistically unrelated to pH, bulk density, total nitrogen, or elevation (Figure 3i–x). Notably, no FD metric exhibited a robust association with elevation, and soil nutrient parameters explained negligible variation in functional structure.

### Species composition patterns along the salinity gradient

**FIGURE 4:**
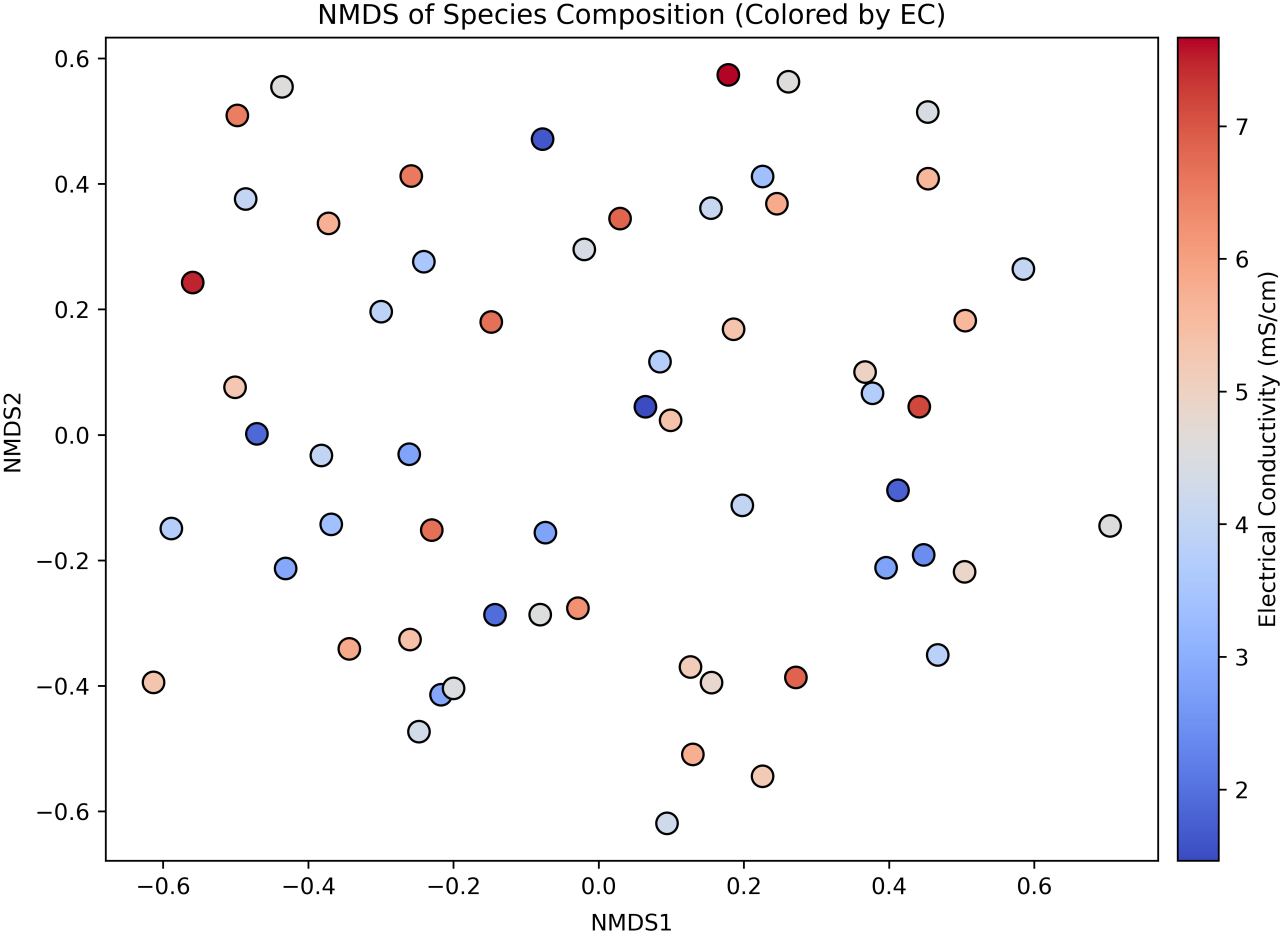
Non‐metric multidimensional scaling (NMDS) ordination of species composition based on Bray–Curtis dissimilarity. Each point represents a plot, with positions reflecting differences in species assemblages. Points are color coded by electrical conductivity (EC, in millisiemens per centimeter) to visualize community variation along the salinity gradient. The ordination was performed using two dimensions with a non‐metric MDS algorithm applied to square‐root‐transformed species abundance data.

NMDS ordination revealed spatial turnover in species composition across the salinity gradient (stress = 0.293; Figure 4). Plot scores displayed continuous variation along the NMDS1 and NMDS2 axes, with no evidence of discrete clustering, suggesting that species turnover occurs along a continuum of abiotic filtering rather than through sharp community boundaries.

Color mapping by EC demonstrated that salinity was spatially structured across ordination space. Plots with high EC values (>6 mS/cm) were concentrated toward the positive NMDS1 axis, whereas low‐salinity plots (<3 mS/cm) were predominantly located along the negative NMDS1 and NMDS2 space. This spatial alignment of salinity values with ordination axes suggests a strong compositional response to soil salinity, consistent with the directional abiotic filtering of salt‐tolerant versus salt‐sensitive species.

The moderate stress value (0.293) indicates a reasonable two‐dimensional representation of Bray–Curtis dissimilarities among plots, capturing the dominant gradients in community composition while acknowledging residual complexity.

### Foliar trait composition shifts along the salinity gradient

**FIGURE 5:**
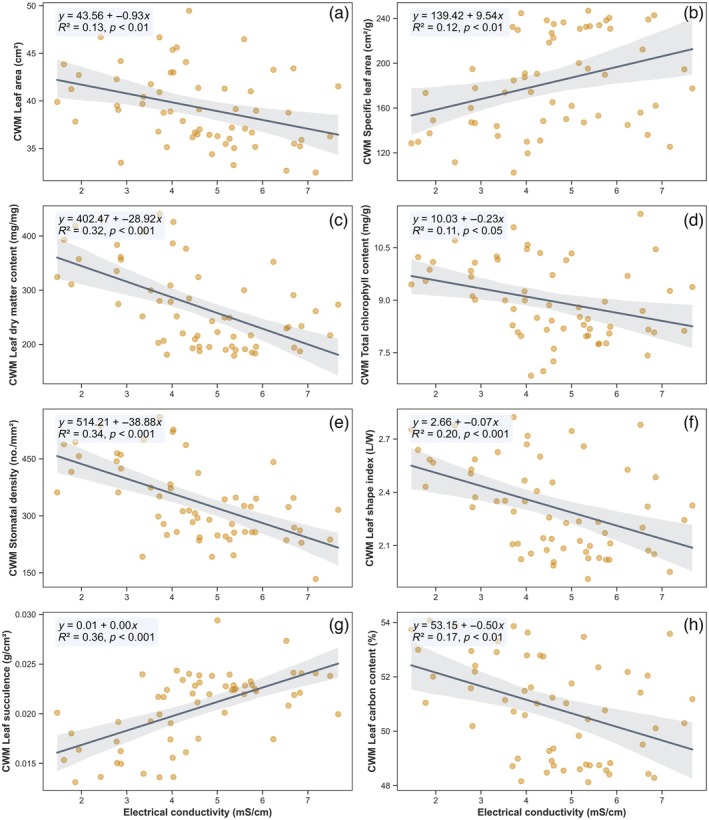
Relationships between electrical conductivity (EC) and community‐weighted mean (CWM) leaf traits. Panels (a)–(h) show linear regressions of EC against CWM leaf area, specific leaf area, leaf dry matter content, total chlorophyll content, stomatal density, leaf shape index, leaf succulence, and leaf carbon content, respectively. Shaded bands indicate 95% CIs, and each panel includes the fitted regression equation, coefficient of determination (*R* ^2^), and *p* value.

Salinity was significantly associated with consistent shifts in CWM values for all eight foliar traits, indicating strong trait–environment filtering across the gradient (Figure 5a–h). Traits indicative of conservative resource‐use strategies increased in more saline plots, whereas traits associated with acquisitive strategies declined.

Specifically, LA (CWM LA) declined significantly with increasing EC (*R* ^2^ = 0.13, *p* = 0.005), reflecting a shift toward smaller leaved species under high salinity (Figure 5a). Conversely, SLA (CWM SLA) increased with EC (*R* ^2^ = 0.12, *p* = 0.007), suggesting thinner leaves in high‐salinity zones (Figure 5b). LDMC (CWM LDMC) showed a strong negative relationship with EC (*R* ^2^ = 0.32, *p* < 0.001), indicating reduced tissue density and structural reinforcement under saline stress (Figure 5c). TC (CWM TC) also declined with EC (*R* ^2^ = 0.11, *p* = 0.011), possibly reflecting impaired photosynthetic potential (Figure 5d). SD (CWM SD) exhibited one of the strongest responses, with a significant decrease under higher salinity (*R* ^2^ = 0.34, *p* < 0.001; Figure 5e), pointing to reduced gas exchange capacity. LSI (CWM LSI) also decreased (*R* ^2^ = 0.20, *p* < 0.001), suggesting rounder leaves in more saline sites (Figure 5f). LS (CWM LS) increased markedly with salinity (*R* ^2^ = 0.36, *p* < 0.001; Figure 5g), highlighting enhanced water‐storage capacity as an adaptive mechanism. Finally, LC (CWM LC) declined significantly with salinity (*R* ^2^ = 0.17, *p* = 0.001; Figure 5h), potentially reflecting reduced lignin or structural compound investment. Each trait exhibited a directionally consistent trend, with all models statistically significant (*p* < 0.05), and the highest coefficients of determination observed for LS and SD.

### Multivariate ordination of trait composition along the salinity gradient

**FIGURE 6:**
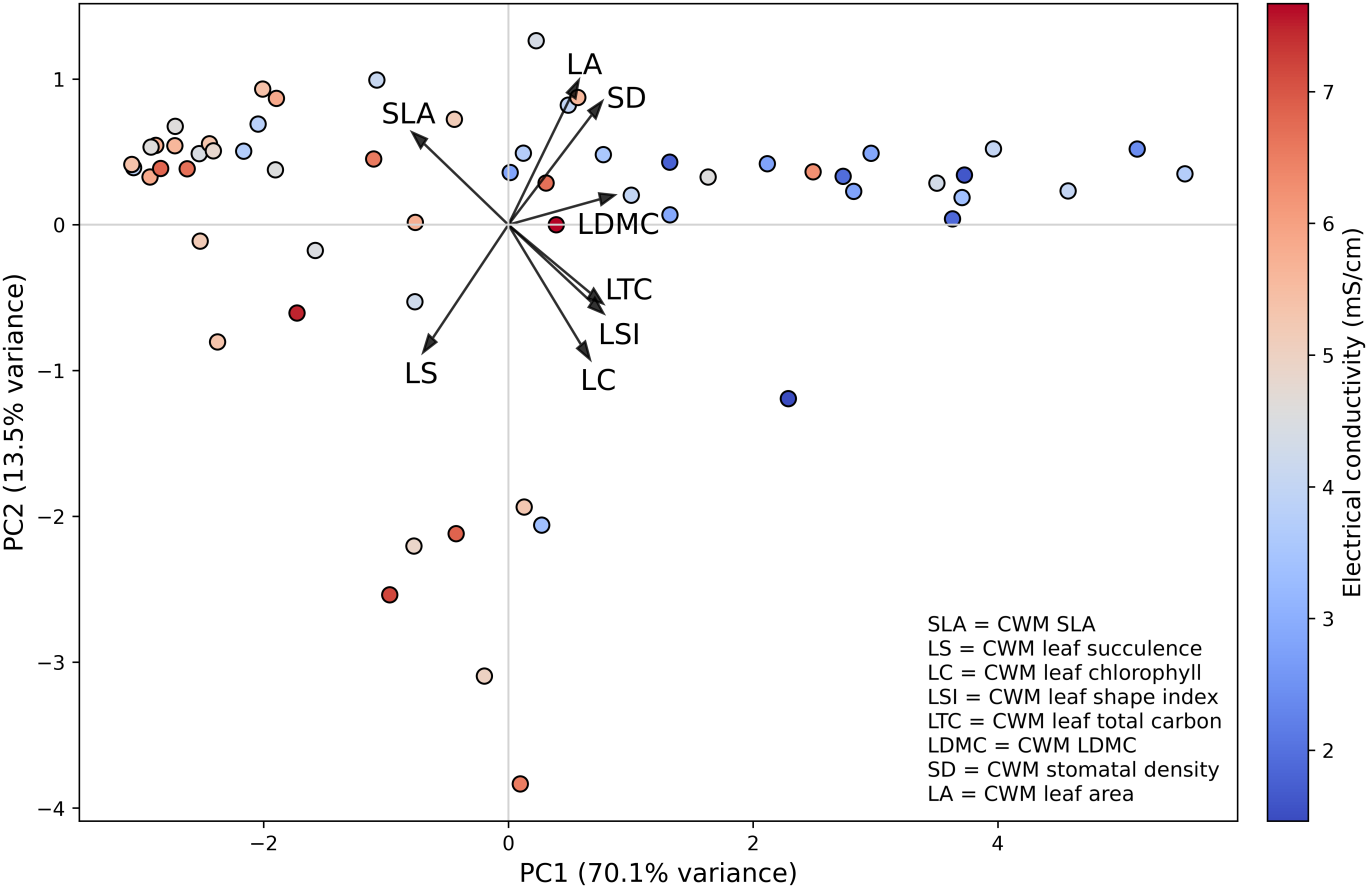
Principal components analysis (PCA) of community‐weighted mean (CWM) leaf traits across plots, with points colored by electrical conductivity (EC). Trait vectors represent loadings of nine CWM traits: Leaf area (LA), specific leaf area (SLA), leaf dry matter content (LDMC), total chlorophyll content (LTC), stomatal density (SD), leaf shape index (LSI), leaf succulence (LS), and leaf carbon content (LC). Axes show the first two principal components, which together explain 83.6% of total variance (PC1: 70.1%, PC2: 13.5%). Point color gradients indicate EC values (in millisiemens per centimeter), highlighting salinity‐associated shifts in multivariate trait composition.

Principal components analysis (PCA) of CWM traits revealed strong covariation among foliar traits across the salinity gradient (Figure 6). The first two principal components together explained 83.6% of the total variance, with PC1 and PC2 accounting for 70.1% and 13.5%, respectively.

PC1 was primarily associated with high positive loadings of LDMC (0.41), SD (0.36), LC (0.37), and LSI (0.37), while exhibiting strong negative loadings for SLA (−0.37) and LS (−0.33). PC2 showed strongest contributions from LA (0.47), SD (0.40), and SLA (0.30), with negative associations for TC (−0.44) and LS (−0.42).

Plots were color‐coded by EC, indicating that variation along PC1 aligned with the salinity gradient. Sites with higher salinity tended to occupy the negative end of PC1, whereas low‐salinity plots clustered on the positive side, reflecting a gradient in trait composition. Trait vectors displayed strong directional separation, suggesting coordinated multivariate shifts in trait expression corresponding to salinity levels.

## DISCUSSION

Our study provides robust evidence that soil salinity functions as a dominant abiotic filter shaping foliar functional trait composition and, to a lesser extent, community‐level FD in the Sundarbans mangrove forest. By integrating fine‐scale trait measurements with FD indices, we observed distinct patterns of trait convergence and declining functional dissimilarity in more saline zones. These results align with predictions from trait‐based community assembly theory (Díaz et al., 2007; Violle et al., 2007), although the observational design limits our ability to directly test the underlying mechanisms.

### FD responses to salinity filtering

In support of hypothesis (i), FD—measured by Rao's quadratic entropy (RaoQ)—declined significantly along the soil salinity gradient, whereas FRic, FEve, and FDiv showed no significant response. The stability of FRic and FEve likely reflects trait redundancy or niche packing, where species with overlapping functional traits coexist despite shifts in dominant strategies, resulting in limited changes in trait volume and evenness. Because RaoQ integrates both trait dissimilarity and relative abundance, it is particularly sensitive to the dominance of functionally similar salt‐tolerant species (Botta‐Dukát, 2005). This supports the abiotic filtering hypothesis, which posits that environmental stressors constrain viable trait combinations, reducing FD (Cornwell & Ackerly, 2009; Spasojevic & Suding, 2012). Similar reductions in RaoQ under salinity stress have been documented in arid rangelands (Rafiee et al., 2022) and saline‐alkali wetlands (Wang, He, et al., 2024), though evidence from mangrove ecosystems has remained limited until now.

Spatially, this reduction in RaoQ was most pronounced in the western Sundarbans, where diminished freshwater inflows elevate soil EC (Akbar Hossain et al., 2021; Sarker et al., 2016). These areas are increasingly dominated by salt‐tolerant generalist species such as *E. agallocha* and *Ce. decandra*, which exhibit similar foliar traits, reinforcing community‐wide functional convergence.

### Biotic structure modulates trait dispersion

Our findings strongly support hypothesis (iii), demonstrating that biotic structure—specifically species abundance and richness—significantly modulates FD independent of salinity. Importantly, species abundance emerged as a key driver, negatively influencing RaoQ and FEve, but positively influencing FDiv. This indicates that increasing dominance by stress‐tolerant species compresses trait evenness and dissimilarity while maintaining divergence among dominant taxa occupying peripheral trait space (Chalmandrier et al., 2015). These results highlight the dynamic interplay between abiotic filtering and biotic interactions in shaping trait distributions.

Species richness exhibited a positive relationship with FRic, confirming that greater richness expands community trait space (Laliberté & Legendre, 2010; Petchey & Gaston, 2002). However, the lack of association between richness and RaoQ or FEve underscores that species number alone does not guarantee higher trait dissimilarity or evenness, reflecting the role of trait convergence and redundancy (Cadotte et al., 2011; Spasojevic & Suding, 2012).

The explicit inclusion of biotic structure in this analysis offers insights often overlooked in trait‐based studies, particularly within mangroves. Prior research has frequently attributed trait convergence solely to abiotic stress, neglecting the mediating role of community composition and competition (Alongi, 2018; Rahman et al., 2021). Our results demonstrate that variation in abundance and richness can significantly alter interpretations of FD metrics, potentially masking the direct effects of abiotic filters. Therefore, future trait‐based research should systematically incorporate these biotic dimensions to disentangle interactive drivers of diversity.

Environmental covariates measured in this study, like soil pH, elevation, total nitrogen, and bulk density, were weak predictors of FD indices. Their limited explanatory power suggests that salinity acts as the primary abiotic constraint in the Sundarbans, overshadowing secondary edaphic influences. Similar dominant effects of salinity have been reported across other coastal ecosystems, confirming its global ecological importance (Alongi, 2018; Meera et al., 2023). The consistent lack of associations with other soil variables further implies that physiological stress from salinity may override subtler soil effects.

Overall, this study emphasizes that species abundance and richness play central roles in modulating trait dispersion under salinity stress. These findings demonstrate the complex interplay between abiotic filtering and community composition and the need for integrated frameworks that consider both in explaining mangrove functional organization.

### Foliar trait composition shifts along the salinity gradient

Consistent with our hypothesis (ii), significant coordinated shifts in CWM foliar traits occurred along the salinity gradient, reflecting directional adaptive responses. Reductions in SD likely represent a physiological adjustment reducing transpiration to conserve water under high salinity (Ball, 1988; Parida et al., 2016). Increasing salinity reduced LA, LDMC, TC, SD, LSI, and LC, while increasing LS and SLA. These coordinated responses indicate strong selection for traits promoting osmotic adjustment and water conservation (Matinzadeh et al., 2022).

Reductions in LA, LDMC, and LSI reflect classic adaptations minimizing water loss by reducing surface area and structural investment while enhancing water‐use efficiency (Tamene et al., 2024). Decreased SD and TC suggest lower photosynthetic capacity and gas exchange, highlighting trade‐offs between carbon gain and stress tolerance (Lovelock & Feller, 2003). Together, these patterns reveal a shift toward conservative metabolic strategies, consistent with the global leaf economics spectrum (Wright et al., 2004).

Although the observed increase in SLA under high salinity seems paradoxical, it likely reflects enhanced LS, which dilutes intracellular salt concentrations and improves osmotic balance (Meera et al., 2023). This plasticity demonstrates fine‐tuned physiological responses enabling survival under extreme salinity. Importantly, these community‐weighted trait signals capture ecosystem‐level reorganization driven by shifting species dominance, rather than species‐level adaptations alone (Muscarella & Uriarte, 2016).

Overall, the coordinated trait responses highlight salinity as the dominant selective force shaping mangrove foliar traits and stress the importance of conserving low‐salinity zones to maintain FD and resilience. Understanding these relationships is vital for predicting ecosystem responses to increasing salinity and informing adaptive management strategies under a changing climate.

### Multivariate trait coordination along salinity gradients

Our PCA ordination revealed clear coordination among foliar traits along the salinity gradient, with the first principal component (PC1) explaining approximately 70% of the total variation. PC1 was strongly aligned with EC, demonstrating that salinity acts as a dominant abiotic filter structuring functional trait composition within mangrove communities. Traits associated with salt tolerance, such as increased LS and higher SLA, were positively correlated with higher salinity, whereas LDMC, SD, and LC exhibited negative associations. These trait combinations underscore coordinated ecological trade‐offs between structural integrity, water retention, and photosynthetic efficiency under salt stress (Hu et al., 2021; Kaleem et al., 2024).

The alignment of multiple trait vectors along PC1 demonstrates coordinated syndromes rather than isolated responses. Elevated succulence and SLA facilitate internal water storage and salt dilution, while reduced LDMC and SD reflect lower structural and gas‐exchange investments (Nguyen et al., 2017). Together, these traits illustrate conservative resource‐use strategies consistent with the global leaf economics spectrum (Wright et al., 2004).

Comparable multivariate coordination has been documented in salt marshes (Lubińska‐Mielińska et al., 2024) and coastal dunes (Ciccarelli et al., 2023). However, our study provides one of the first community‐level analyses explicitly linking foliar trait syndromes to continuous salinity gradients in mangroves. These findings advance understanding of mangrove community assembly and offer a predictive basis for assessing resilience under intensifying climate‐driven salinization (Muscarella & Uriarte, 2016; Sarker et al., 2024).

### Implications for forest management and future research directions

The observed declines in FD and directional trait shifts with rising salinity have critical implications for mangrove management and resilience. Salinity‐driven abiotic filtering, intensified by freshwater reduction and sea‐level rise, may homogenize communities by favoring a limited suite of highly salt‐tolerant species. Such homogenization risks eroding functional complexity and weakening key ecosystem services, including carbon sequestration, shoreline protection, and biodiversity maintenance (Alongi, 2018; Sarker et al., 2024).

Conservation priorities should therefore focus on protecting and restoring low‐salinity zones, which retain higher functional and structural diversity (Rahman et al., 2021). Conservation efforts should specifically target species exhibiting distinct trait combinations that promote FDis, such as *H. fomes*, which is already experiencing declines in areas of elevated salinity (Rahman et al., 2025). In particular, restoration strategies may consider targeted planting of *H. fomes* and other freshwater‐associated species in low‐salinity zones, where their trait profiles are most compatible with local conditions, thereby enhancing ecosystem resilience. Assisted regeneration and targeted planting guided by trait‐based frameworks can help sustain or enhance mangrove functionality (Rodríguez‐Rodríguez et al., 2021; Thivakaran, 2017).

This study underscores the value of trait‐based approaches as a scalable tools for anticipating ecological responses to salinization. Integrating detailed trait data with spatial salinity maps enables managers to move beyond species inventories toward predictive models of resilience and function (Muscarella & Uriarte, 2016; Violle et al., 2007). Future research should pair hydrological management—such as regulated freshwater inflows and sediment control—with remote sensing of key foliar traits (e.g., SLA and chlorophyll) to monitor ecosystem health at broader scales (Lewis, 2005; Su et al., 2024). In addition, remote sensing of foliar traits such as SLA and chlorophyll content provides a promising monitoring tool to track mangrove health and functional responses to salinity at broader spatial scales, complementing plot‐based trait surveys.

Expanding trait datasets to include root and hydraulic traits, along with reproductive strategies, will further strengthen predictive trait–environment models (Madhavan et al., 2024; Meera et al., 2023). Moreover, understanding how geomorphic and hydrological variables interact with salinity to shape trait expression will be crucial for effective, long‐term restoration planning. Given the global importance of mangroves in climate mitigation and coastal protection (Alongi, 2008; Uddin et al., 2023), these trait‐informed management practices offer valuable insights for similar deltaic ecosystems worldwide.

Ultimately, linking fine‐scale trait data to landscape‐scale conservation decisions represents a key frontier in climate‐adaptive mangrove management. Such integration will be essential for maintaining the ecological integrity, functionality, and adaptive capacity of mangrove forests facing future environmental change.

## CONCLUSION

This study shows that salinity is a key ecological filter that drives foliar trait convergence and lowers FD in the Sundarbans mangrove forest. The dominance of stress‐tolerant traits reflects adaptive trade‐offs in water conservation, tissue structure, and osmotic balance. Biotic factors, including species abundance and richness, also influence trait variation, highlighting the complex interactions between environmental stress and community composition. By linking community‐weighted traits with fine‐scale salinity patterns, this research reveals how climate‐driven salinization reshapes mangrove ecosystems. These findings strengthen trait‐based ecological understanding and offer practical guidance for conservation. Protecting low‐salinity zones and using trait‐based indicators in management will be essential to maintain the resilience, biodiversity, and carbon storage capacity of mangroves in a changing climate.

## AUTHOR CONTRIBUTIONS

Md Rezaul Karim and Shekhar R. Biswas conceived the ideas and designed the methodology. Md Rezaul Karim, Mohammed A. S. Arfin‐Khan, and Sharif A. Mukul collected the data. Md Rezaul Karim and Nabanita Karmaker analyzed the data and led the writing of the original draft. Md Rezaul Karim also developed software and curated data. Mohammed A. S. Arfin‐Khan, Tanjena Khatun, Md. Shamim Reza Saimun, Sanjeev K. Srivastava, Sharif A. Mukul, and Fahmida Sultana critically reviewed and edited the manuscript. Mohammed A. S. Arfin‐Khan, Fahmida Sultana, Sanjeev K. Srivastava, and Sharif A. Mukul acquired funding. Mohammed A. S. Arfin‐Khan supervised the study. All authors contributed critically to drafts and approved the final publication.

## CONFLICT OF INTEREST STATEMENT

The authors declare no conflicts of interest.

## Supporting information


Appendix S1.

## Data Availability

Data (Karim, 2026) are available in Figshare at https://doi.org/10.6084/m9.figshare.31244818.
