# Supplementary material for: Trait‐based evidence of salinity‐induced functional diversity loss in mangroves: Implications for ecosystem resilience
Source: Ecol Appl. 2026 Feb 24;36(1):e70191. doi: 10.1002/eap.70191 (PMC12931479; doi:10.1002/eap.70191)
Supplement: Supplementary file 1 — Appendix S1. [file EAP-36-e70191-s001.pdf]

Appendix S1

Trait-based evidence of salinity-induced functional diversity loss in mangroves: Implications for ecosystem resilience

Md Rezaul Karim, Nabanita Karmaker, Shekhar R. Biswas, Md. Shamim Reza Saimun, Sharif A. Mukul, Tanjena Khatun, Fahmida Sultana, Sanjeev K. Srivastava, Mohammed A. S. Arfin-Khan

Ecological Applications

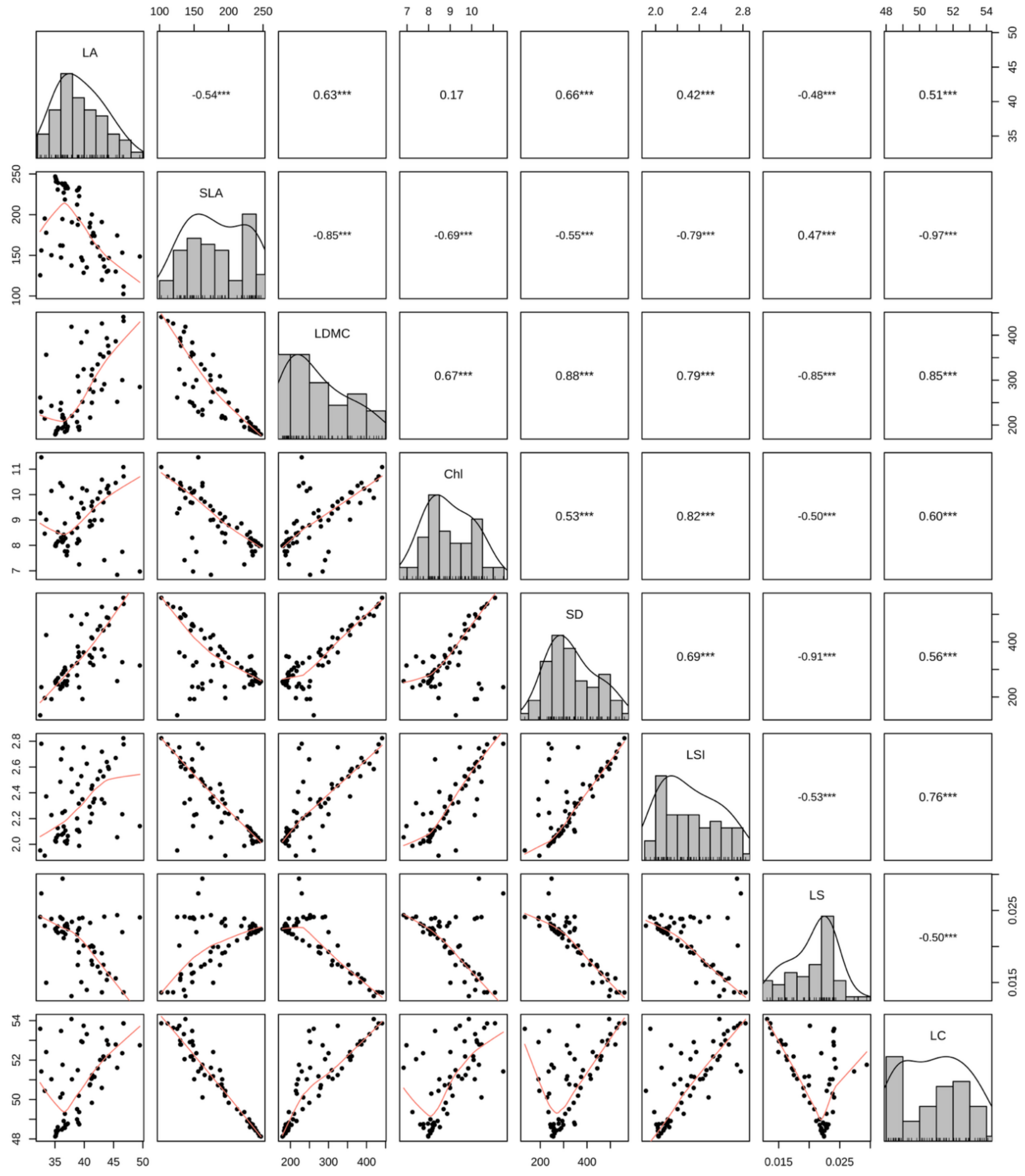

Figure S1 (caption on next page)

Figure S1: Pairwise relationships among eight community-weighted mean (CWM) leaf traits. Variables include leaf area (LA), specific leaf area (SLA), leaf dry matter content (LDMC), total chlorophyll content (Chl), stomatal density (SD), leaf shape index (LSI), leaf succulence (LS), and leaf carbon content (LC). Lower panels display bivariate scatterplots with loess smoothers; diagonal panels show histograms with density curves. Upper panel's report Pearson correlation coefficients, with significance levels indicated ($p < 0.05$: *, $p < 0.01$: **, $p < 0.001$: ***).

Table S1: Description of measured foliar functional traits.

Traits	Mathematical Equation/ Tools Used	Unit	References
Leaf area (LA)	Mobile application Petiole: Plant leaf area meter	cm^2	(Singh et al. 2021)
Specific Leaf Area (SLA)	$SLA = \frac{LA}{\mathrm{LDM}}$ Here, LA = Fresh leaf area (cm^2); LDM = Leaf oven-dry mass (g)	cm^2g^{-1}	(Garnier et al. 2001)
Leaf Dry Matter Content (LDMC)	$LDMC = \frac{Dry\ Weight}{\mathrm{Fresh\ Weight}}$	$mg.mg^{-1}$	(Garnier et al. 2001)
Leaf Shape Index (LSI)	$LSI = \frac{Leaf\ Length}{\mathrm{Leaf\ Width}}$	-	(Schrader et al. 2021)
Leaf Succulence (LS)	$LS = \frac{LGM - LDM}{\mathrm{LA}}$ Here, LGM = Leaf green mass (g); LDM = Leaf oven-dry mass (g); LA = Fresh leaf area (cm^2)	gcm^{-2}	(Ogburn and Edwards 2012)
Stomatal density (SD)	Nail polish imprint method; counted via digital microscope	stomata·mm^{-2}	(Ceulemans et al. 1995)
Total chlorophyll content (TC)	Acetone extraction (80% acetone) and spectrophotometry	mgg^{-1}	(Ritchie 2008)
Leaf carbon content (LC)	C = (100 − Ash %) × 0.58; measured post ignition at 600°C	% (dry mass)	(Shaw 1959)

References

Ceulemans, R., L. Van Praet, and X. N. Jiang. 1995. Effects of CO_2 enrichment, leaf position and clone on stomatal index and epidermal cell density in poplar *(Populus)*. New Phytologist 131:99–107.

Garnier, E., B. Shipley, C. Roumet, and G. Laurent. 2001. A standardized protocol for the determination of specific leaf area and leaf dry matter content. Functional Ecology 15:688–695.

Johnson, D., J. Voorhis, and S. Porder. 2025. Life cycle emissions associated with vault storage of wood cleared for fire management in the Western United States. Carbon Balance and Management 20:26.

Ogburn, R. M., and E. J. Edwards. 2012. Quantifying succulence: a rapid, physiologically meaningful metric of plant water storage. Plant, Cell & Environment 35:1533–1542.

Ritchie, R. J. 2008. Universal chlorophyll equations for estimating chlorophylls a, b, c, and d and total chlorophylls in natural assemblages of photosynthetic organisms using acetone, methanol, or ethanol solvents. Photosynthetica 46:115–126.

Schrader, J., P. Shi, D. L. Royer, D. J. Peppe, R. V. Gallagher, Y. Li, R. Wang, and I. J. Wright. 2021. Leaf size estimation based on leaf length, width and shape. Annals of Botany 128:395–406.

Shaw, K. 1959. Determination of organic carbon in soil and plant material. Journal of Soil Science 10:316–326.

Singh, J., A. Kumar, and L. Singh. 2021. Performance of the petiole mobile application on the leaf area estimation as varied with calibration height. The Pharma Innovation 10:337–341.
